# Sustainable worm control in ruminants in Europe: current perspectives

**DOI:** 10.1093/af/vfae033

**Published:** 2024-10-14

**Authors:** Johannes Charlier, Laura Rinaldi, Eric R Morgan, Edwin Claerebout, Dave J Bartley, Smaragda Sotiraki, Marcin Mickiewicz, Maria Martinez-Valladares, Natascha Meunier, Tong Wang, Alistair Antonopoulos, Helena C de Carvalho Ferreira

**Affiliations:** Kreavet, Kruibeke, Belgium; Department Veterinary Medicine and Animal Production, University of Napoli Federico II, Naples, Italy; Institute for Global Food Security, Queen’s University Belfast, Belfast, UK; Laboratory of Parasitology, Faculty of Veterinary Medicine, Ghent University, Merelbeke, Belgium; Moredun Research Institute, Edinburgh, UK; Veterinary Research Institute, Ellinikos Georgikos Organismos (HAO)-DIMITRA, Thessaloniki, Greece; Toinen Pro Art Fundacja, Zduny, Poland; Division of Veterinary Epidemiology and Economics, Institute of Veterinary Medicine, Warsaw University of Life Sciences-SGGW, Warsaw, Poland; Departamento de Sanidad Animal, Instituto de Ganadería de Montaña (CSIC-Universidad de León), León, Spain; Animal Health Ireland, Carrick-on-Shannon, Ireland; Kreavet, Kruibeke, Belgium; Kreavet, Kruibeke, Belgium; Flanders Research Institute for Agriculture, Fisheries and Food, Merelbeke, Belgium

**Keywords:** anthelmintic resistance, helminths, implementation, integrated control, nematode, ruminants

**Implications**

- Anthelmintic resistance is an escalating problem in Europe and the environmental consequences (soil and aquatic health) related to anthelmintic use are an increasing matter of concern.
- Several sustainable worm control (SWC) practices are available now. These include the increased use of diagnostics and decision support enabling a targeted use of anthelmintics. Complementary control measures, referred to as the “Basket of Options”, include plant-based control, grazing management, nematode-destroying fungi, and selective breeding and can also reduce the need for anthelmintic use. Their use is more complex than the simple use of anthelmintics and their uptake has remained relatively low.
- Equipped by recent studies on the barriers to and drivers of uptake of SWC practices, it is now time to develop a Community of Practice across Europe, involving all relevant stakeholders at local, national, and European levels to achieve SWC together.

## Introduction

Cattle, sheep, goats, and their associated industries are a vital component in the development of rural areas and sustainable land use strategies in Europe. With 77 million bovines and 74 million sheep and goats in the EU (Eurostat, 2023), ruminant production is deeply linked to European culture. All European farmed ruminant populations with outdoor access are exposed to parasitic worm (helminth) infections and these remain an important constraint on ruminant productivity. They cost the sector an estimated over €1.8 billion a year, with 80% of this due to production losses and 20% due to treatment costs (Charlier et al., 2020). Worm infections are sensitive to weather conditions and the changing climate, can severely impact animal welfare, and lead to an increase in greenhouse gas emissions from parasitized livestock (Charlier et al., 2017; Houdijk et al., 2017). Reducing the burden of helminth infections in livestock is thus an actionable contribution to the United Nation’s sustainable development goals and the EU’s long-term strategy to reduce greenhouse gas emissions from the agricultural sector by 49% by 2050.

Current worm control relies on the regular administration of anthelmintic drugs. However, in a recent meta-analysis of European data aggregated since 2010, the average prevalence of anthelmintic resistance (AR) to the 3 major anthelmintic drug classes ranged between 48% and 86% (Rose Vineer et al, 2020). Cases of cross-resistance and multidrug resistance are increasingly reported (Bordes at al., 2020). Thus, a report from the World Organisation for Animal Health (WOAH, formerly OIE) warns of the urgent need for responsible and prudent use of anthelmintics to limit the development of AR in grazing livestock (WOAH, 2021). In Europe, stakeholders, including the European Farmers and Agri-Cooperatives (Copa-Cogeca), the Federation of Veterinarians of Europe, and the animal health industry (AnimalhealthEurope) have recognized the need to take action to ensure the responsible use of anthelmintics in food-producing animals through the European Platform for Responsible Use of Medicines in Animals (EPRUMA, 2019).

Research on sustainable worm control (SWC) practices has been occurring for a long time in Europe. Now, it is time to move from research to implementation. This article will lay down the vision to build a European Community of Practice (CoP), over the coming years, supported by the novel Horizon Europe Thematic Network “SPARC—Sustainable Parasite Control in ruminants”.

## A Tradition of Research on SWC in Europe

There is a long tradition of research toward improved worm control in Europe thanks to EU funding (e.g., FP6, FP7, ERA-NETs, Horizon 2020, and Horizon Europe). One of the foundational projects was the EU-funded PARASOL consortium (running from 2006 to 2009), the first transnational project in Europe, and beyond, as the consortium also included partners from Africa. The project recognized that while concerns around the sustainability of helminth control in ruminant livestock had been building globally for well over a decade (Waller, 1993), the application of SWC was critically dependent on finding pragmatic methods for farms in Europe. PARASOL built on refugia-based approaches (Leathwick et al., 2006; Van Wyk et al., 2006) and further developed them through concepts of anthelmintic targeted treatments (TT) and targeted selective treatments, improved in vivo and in vitro tests for detection of AR, and worked on optimizing efficacy and bioavailability of anthelmintic compounds (Vercruysse et al., 2009). During the same period, the DELIVER project addressed the growing problem of liver fluke disease in Europe. The project improved knowledge on *Fasciola hepatica* epidemiology, the genetics of different isolates, and vaccine studies to design effective and sustainable control strategies. Control of both parasitic helminths (nematodes and liver fluke) was integrated into the GLOWORM (2012 to 2014) and PARAVAC (2011 to 2015) projects. GLOWORM developed high-throughput and multiplex diagnostic methods, models predicting parasite infections under climate change conditions, and sustainable control strategies (Rinaldi et al., 2015), whereas PARAVAC consolidated research on vaccines against helminth infections (Matthews et al., 2016). All the above networks allowed for the development of intense research interactions on worm control between European actors. These networks were further consolidated and extended via the creation of the Livestock Helminth Research Alliance in 2014 (Charlier et al., 2017) and the COST Action COMBAR (2017 to 2022; Charlier et al., 2022), resulting in a network of over 200 researchers from across Europe and from various specific disciplines working together to find solutions for the problem of AR. COMBAR was the first project placing emphasis on using more economic and social sciences in the development of SWC strategies, recognizing the central importance of producer behavior (McArthur and Reinemeyer, 2014). It also called for the development of multi-actor approaches involving veterinarians, farmers, pharmaceutical industry, and research bodies to create awareness and cocreate solution paths in mitigating the escalating spread of AR (Höglund et al., 2021). Therefore, SPARC was developed as a natural evolution and culmination of the previous 2 decades’ work to make a pan-European multi-actor movement on sustainable parasite control a reality.

## Developing a CoP

**Figure 1.**
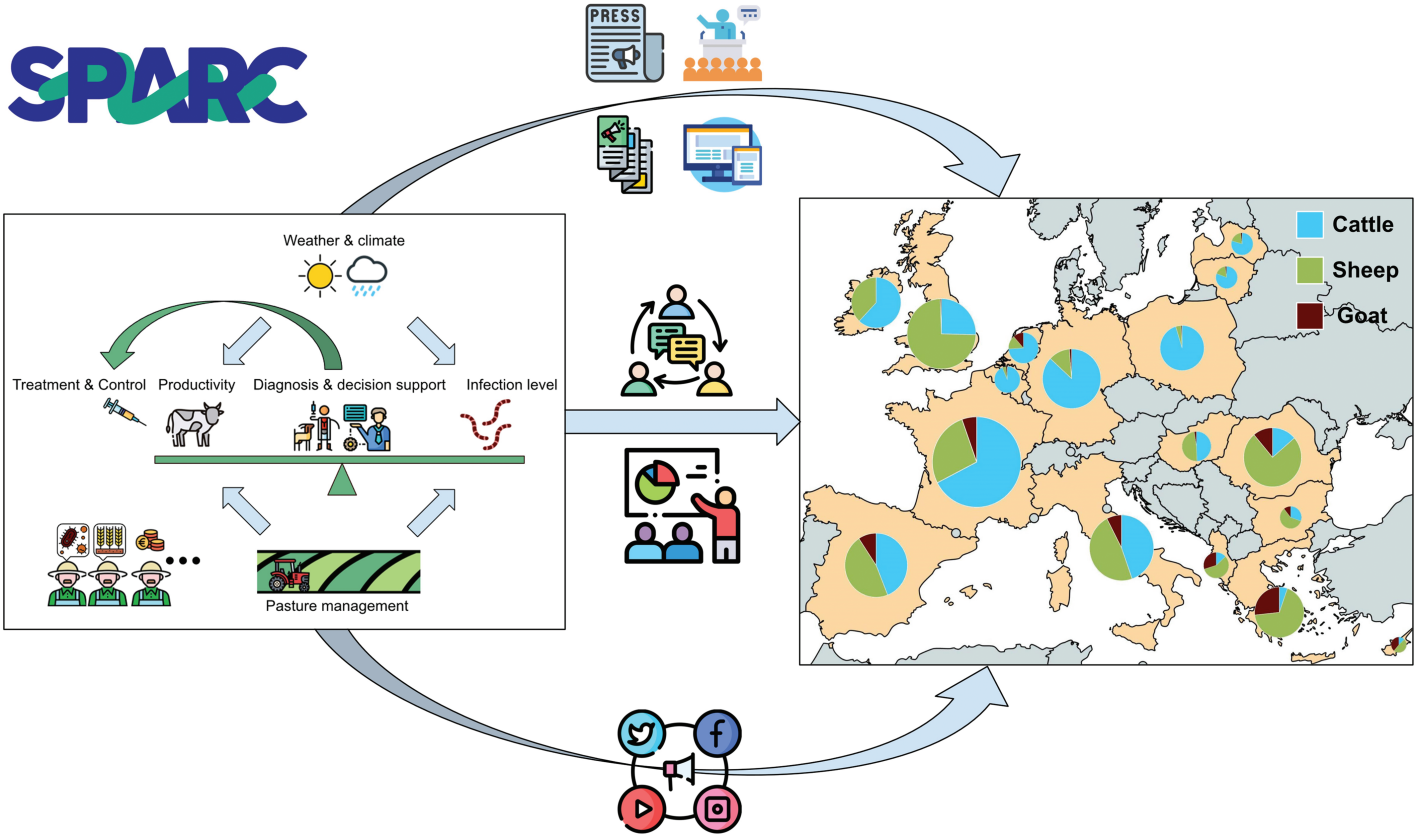
In SPARC, SWC strategies will be translated and promoted into user-friendly advice/guidelines through multiple approaches across Europe. Regional and production type-specific adaptation of these approaches will be applied.

SPARC aims to establish a collaborative network across Europe, with its vast diversity in ruminant livestock production systems (See Text Box), involving various stakeholders to enhance the sustainability, efficiency, and resilience of ruminant livestock farms. The project focuses on curbing the escalating threat of AR and aims to achieve 3 primary objectives: enhancing animal health and welfare, improving economic performance, and promoting environmental sustainability within the sector (Figure 1).

**Figure 2.**
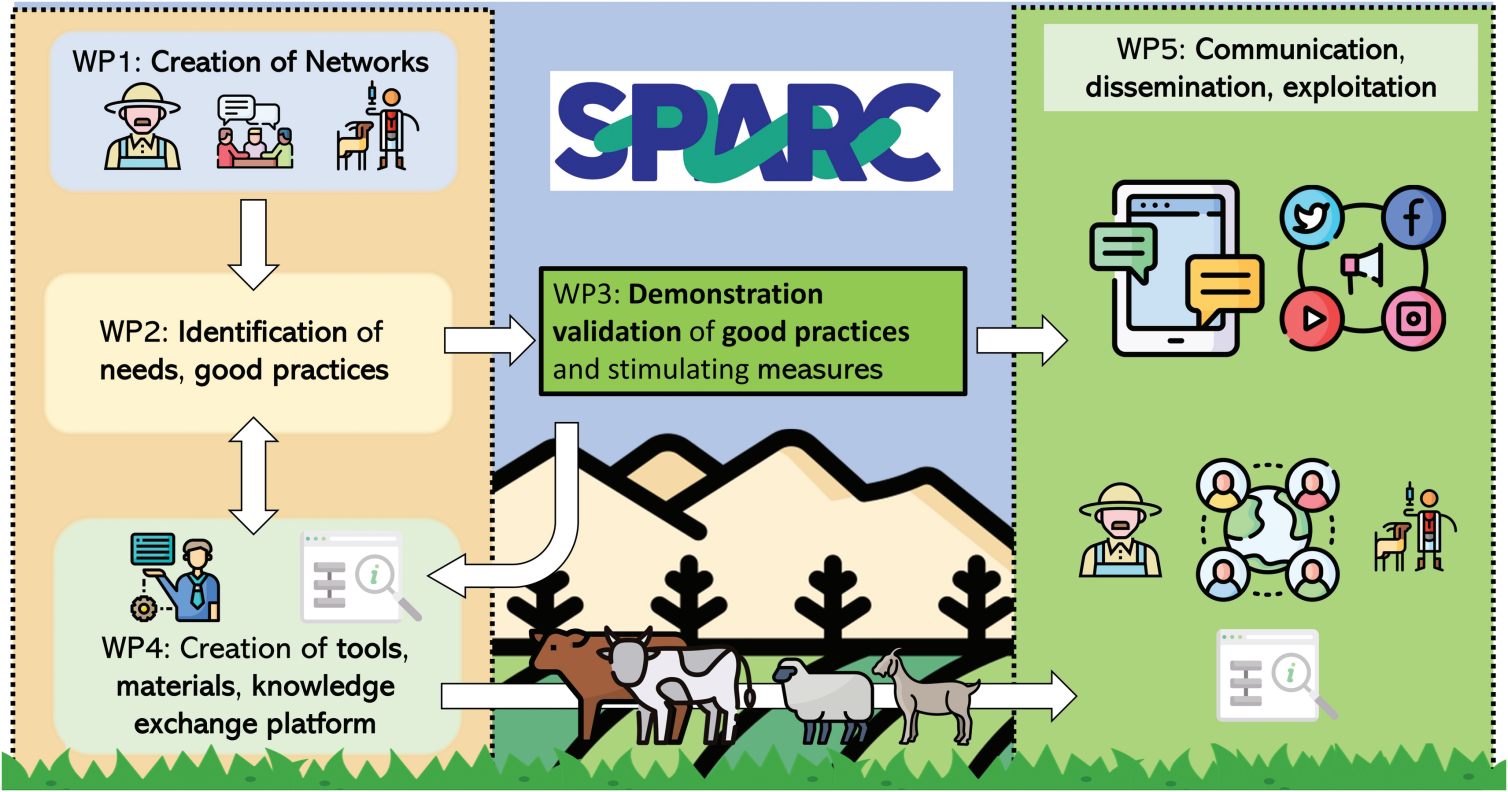
Summary of workflow and interaction of the SPARC project work packages

To achieve its goals, SPARC will engage in horizontal knowledge exchange, drawing insights from farmers, veterinarians, advisors, and industry partners to identify and disseminate cost-effective, practical, stakeholder-driven solutions for sustainable ruminant production (Figure 2).

The project will establish national and regional stakeholder networks across participating countries, leveraging existing networks of partner institutions, and recruiting additional participants to form a CoP. The CoP will revolve around 3 central actor groups: farmers, veterinarians, and farm advisors. These groups collaborate with partners and associated organizations, including governmental agencies, universities, research institutes, technical and pharmaceutical companies, and farmer organizations. This CoP will identify and disseminate best practices for SWC. Stakeholder needs are being assessed through focus groups and selected control strategies are being implemented in pilot farms in 17 countries. The impact of the control strategies on animal productivity, welfare, economic returns, and anthelmintic efficacy will be monitored.

Simultaneously, SPARC is developing a knowledge exchange platform (KEP) populated with insights gathered from the focus groups, the pilot farms, and other activities from associated organizations. The knowledge will then be disseminated through regional CoP subnetworks using multimedia info-packs in different languages with a specific emphasis on overcoming external obstacles encountered during the trial implementation.

**Embracing diversity in systems and approaches**

“United in diversity,” the motto of the EU reflects the diversity in the cultures, traditions, and languages across the European continent. This diversity is equally a characteristic of the European livestock systems and hence the SWC strategies that are practical, feasible, and fit with longstanding traditions. Below we want to show this diversity through a few examples that underline the situation and SWC activities in different countries.

*Ireland* is a predominantly grass-based livestock system, with a temperate, high-rainfall climate, and largely seasonal breeding, particularly in the prominent dairy industry. The extensive reliance on grazing translates to livestock farming that is heavily impacted by helminth infections. Additionally, resistance in both sheep and cattle roundworms and liver fluke is increasingly concerning for the livestock industry. Partly due to this resistance risk, Ireland is currently in a transition period where antiparasitic medicines will require a veterinary prescription, having previously been exempt. This has resulted in a focus on best practice messaging and prudent use of anthelmintics from advisory bodies such as Teagasc and Animal Health Ireland, who facilitate a working group of parasite experts; as well as the establishment of an industry Antiparasitic Resistance Stakeholder Group. Examples of promoted measures include a Targeted Advisory Service on Animal Health focusing on parasite control (Meunier, 2023) and the ongoing development of best practice guidelines. Despite these recent initiatives, like elsewhere, changing behavior to sustainable measures can be slow. Excluding small farms, more than half of Irish farmer households have off-farm employment, 30% of farmers are of pensionable age and an average of one-third of sheep and cattle-rearing farms are economically vulnerable (Teagasc, 2023). Therefore, interventions that are time and cost-effective, with minimal management, are required if a majority of farmers are to adopt these behaviors.

*Poland* is the 5th largest (cattle) milk producer of the EU and 12th largest globally. However, despite the large cattle population, data on the prevalence of AR in this species are unavailable. Knowledge about resistance to anthelmintics among veterinarians, farm advisors, and farmers is low. The method of anthelmintic prevention in cattle herds depends largely on the knowledge and experience of the veterinarian and the financial resources of the owner. The length of the milk withdrawal period is crucial in deciding on the use of anthelmintics, which is why in recent years, drugs with 0 d or a short milk withdrawal period have been used much more often. A common practice is to treat animals with anthelmintics twice a year (at the beginning and end of the grazing season). Data, knowledge, guidelines, and training on SWC practices are not widely available. Knowledge on the prevalence of AR and SWC practices in small ruminants is much better than for cattle (Mickiewicz et al., 2021). The main issue in goats is a lack of anthelmintic agents registered for this species. Moreover, it is common practice to extrapolate doses of anthelmintics from cattle or sheep to goats, which results in the underdosing of goats. Due to the increase in AR, the implementation of SWC practices in goat herds has significantly increased. Nevertheless, regardless of the grazing ruminant species, the lack of generally available knowledge and recommendations regarding SWC practices is still a limitation for farmers, farm advisors, and veterinarians.

The *United Kingdom* has important breeding sheep (14 million heads), dairy cow (1.8 million), and beef cattle (1.3 million) populations. The industries encompass a wide range of scales and landscapes from hobby farmers, crofters through to pedigree/commercial enterprises. Spatial and temporal differences in parasite exposure are common across the United Kingdom with climatic changes making predicting the timing of treatments more difficult. Control of parasites is heavily reliant on the chemotherapeutic or prophylactic use of anthelmintics across the board with alternative strategies of control increasing in popularity. One of the reasons for this is the increasing prevalence of AR in both sheep and cattle parasites (Bartley et al., 2021). Unlike many other regions of Europe, medicines such as anthelmintics can be sourced from suitable qualified persons as well as pharmacists and veterinarians. Numerous grazing strategies are employed across the sector including co-grazing, rotational grazing, cellular grazing as well as communal grazing. Going forward areas such as targeted treatments based on diagnostics/pathophysiological markers, breeding for resistance/resilience/tolerance, and nutrition/grazing management are likely to become increasingly important with a need to optimize timings to identify issues, reduce AR development and spread, minimize environmental impacts, and improve biodiversity whilst maintaining a productive industry.

With over 7 million sheep and 3 million goats, *Greece* accounts for 14% and 31% of Europe’s sheep and goat population, respectively. Greece is the only country where sheep (39.4%) and goats (16.1 %) constitute the highest share of animals within a national livestock population. The specific landscape and climate conditions of the country have favored the development of traditional sheep and goat farming systems which are highly important for the local economy and society and deeply linked to the local culture. Diagnostics are not routinely used to inform on the timing or frequency of deworming, resulting in inefficient and often too intensive application of deworming (Lianou et al., 2023). There is a lack of registered anthelmintics for goats, resulting in the use of anthelmintics at dose rates for sheep.

In *Spain*, the ruminant livestock population comprises 6.5 million bovines, 14 million sheep, and 2.5 million goats. Spain contributes approximately 11% and 5% of the beef and milk produced in the EU, respectively, has the largest sheep population and the second largest goat population (after Greece) in the EU. Due to the extensive nature of a significant part of the sector and its presence where no other economic activities are possible, the sheep and goat sector plays a crucial role in territorial structuring, environmental protection, and job creation in rural areas. Spain has a high diversity in agro-climatic environments which, in conjunction with human action, have configured a great variety of agricultural systems. The main extensive systems are concentrated in the north and center of the country, where animals are more dependent on grazing and are therefore at greater risk of infection by helminth parasites. Administration of anthelmintic drugs is the mainstay of control and AR is present. However, studies on the prevalence of AR have not been carried out throughout the national territory and have focused mainly on sheep, with few data available for cattle and goats (Martínez-Valladares et al., 2020). There have been small initiatives in targeted selective treatment approaches in sheep in northwestern and central Spain using the BCS or live body weight and FECs as treatment indicators. Also, the use of phenotypic and genotypic factors for the selection of animals resistant to gastrointestinal nematode infection has been trialed (Pérez-Hernández et al., 2023). The Spanish Ministry of Agriculture is committed to defend native breeds and bring added value to the rural environment and livestock production. This can act as a spearhead for developing sustainable and quality production models, including SWC practices.

## Basket of Options

There is much scope to improve the ways anthelmintics are used and decrease the dependency of livestock sectors on them by increasing the use of complementary control approaches like pasture management, bioactive forages, and vaccines. This would have the additional benefit of limiting the leakage of anthelmintic residues into the environment, which can impact coprophagous invertebrates (Lumaret et al., 2012) and potentially soil and water health. Because anthelmintics are relatively cheap and easy to administer, however, it is easy for farmers to treat frequently and prophylactically. Moving away from this anthelmintic-first paradigm requires adaptability and recognition that the new tools are not like-for-like replacements for anthelmintics. Rather, they should be used in a complementary way alongside each other and alongside anthelmintics, in a balanced strategy that fits the aims and local circumstances on each farm.

To be successful and sustainable, SWC practices must therefore take into account many factors, which can differ between sectors, regions, and individual farms. This leads to a dilemma, whereby messaging around the need for change should ideally be simple, intuitive, and consistent; yet the variation in context and in farmers’ capacity to adopt specific interventions prohibits a one-size-fits-all approach. Addressing this problem, SPARC applies a basket of options strategy, building on Krecek and Waller (2006) and which will be implemented in the KEP (www.wormsparc.com). Within the “basket” are different tools, which can be chosen to match availability and circumstance, and applied to groups or individual animals according to need, based on epidemiological conditions and information from diagnostic monitoring.

**Figure 3.**
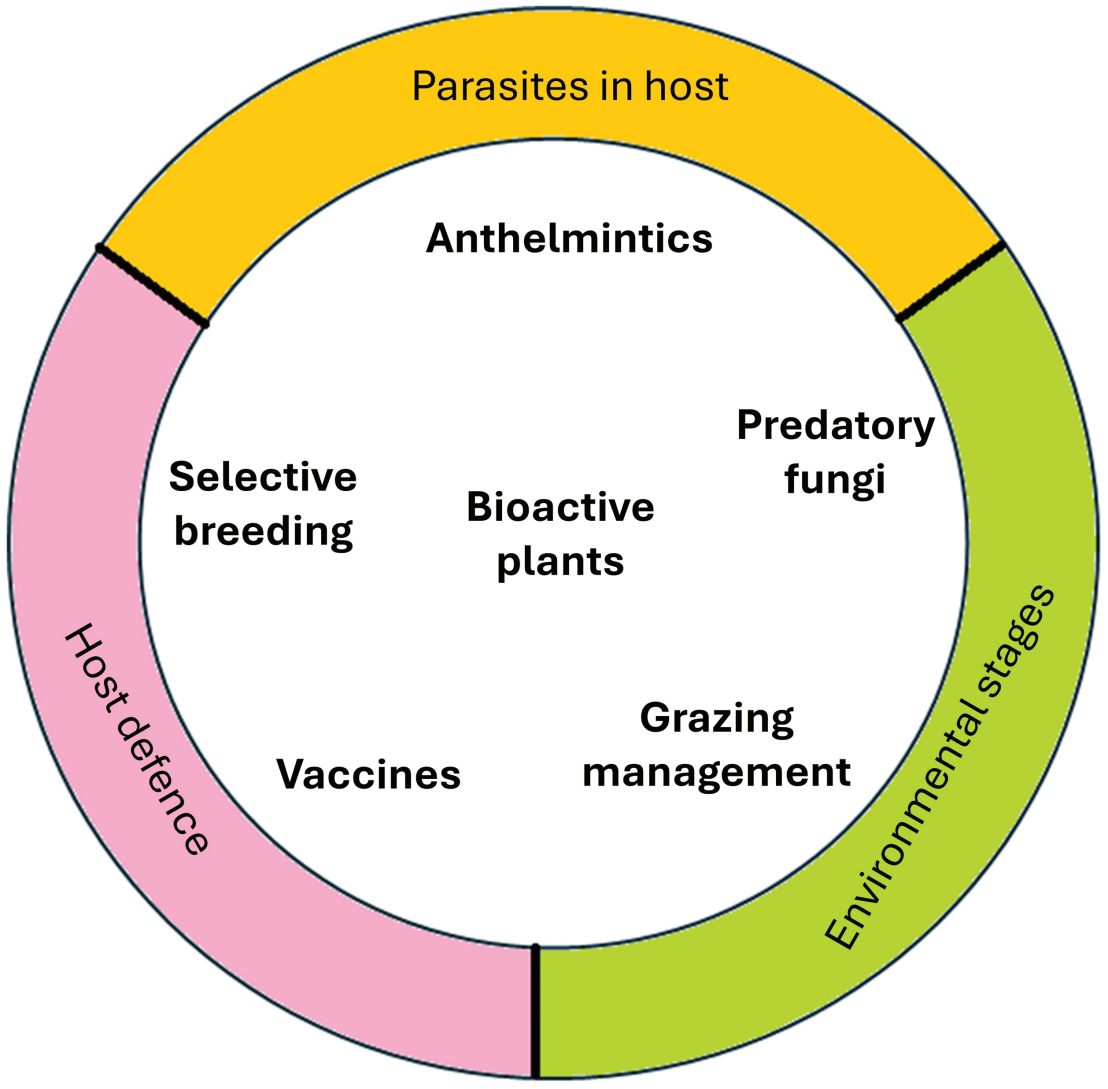
Tools within the “basket of options,” which can be applied for bespoke farm solutions in support of SWC. Modes of action on different elements of the host–parasite system (outer ring) are indicated by position.

Tools within the basket variously target parasites within the host or their free-living stages in dung or on pasture; or seek to support the host’s resistance or resilience to infection (Figure 3). Beyond direct interventions against free-living stages, grazing management makes use of the weather effects on their development and survival, such that animals are moved to avoid contaminating pastures or to evade high levels of pasture infectivity, returning at a safer time. Bioactive plants, meanwhile, can have actions that inhibit parasites directly inside the host or in dung or support host nutrition and immunity (Hoste et al. 2015) and can be delivered as feed supplements or enriched pasture swards. Predatory fungi fed to animals as spores hatch in the dung to suppress the production of infective stages but do not alter adult worm burdens (Fernández et al. 2023); while vaccination acts through host immunity to suppress infection pressure (Claerebout and Geldhof, 2020). Significant amounts of work on “novel approaches” to parasite control in sheep have been undertaken but significantly less has been completed in cattle. Care needs to be taken when extrapolating findings from sheep studies to cattle and vice versa due to the inherent differences in areas such as host biology/immunology, treatment options, parasite and grazing management practices, and labor involved with handling cattle. Table 1 compares the availability of different tools in the sheep and cattle sectors. SPARC recognizes that farmers and their advisors need clear signposting of the different options, how they act, expected effects and—importantly—limitations, delivering this in a format that allows rapid shortlisting of best-fit options and sensible application.

**Table 1.** Availability of different SWC tools in sheep and cattle sectors

Tools	Sheep	Cattle
Targeted and targeted selective treatment approaches	✓ R, C*	✓ R, C*
Grazing/pasture management	✓ R, C*	✓ R, C*
Vaccines	✓ R, C	x
Selective breeding	✓ R, C*	✓ R, C*
Bioactive plants	✓ R, C*	✓ R, C*
Predatory fungi	✓ R, C	✓ R, C
Diagnostics (e.g., FECs)	✓ R, C*	✓ R, C

Abbreviations: ✓, available; x, unavailable; R, research tool; C, commercially available; C*, widely commercially available.

## Diagnostic Options

Diagnosis of worm infections, anthelmintic efficacy, and AR are a benchmark for providing valuable information for effective and sustainable control of helminths. However, in practice, the promotion of diagnostic tools in the development of an SWC program depends on several factors, including ease of use, cost, and informative value (Rinaldi et al., 2022).

The proper understanding and interpretation of diagnostic tests are crucial for optimal decision-making. Relying on a single test result can be misleading, and in most cases, multiple factors need to be considered before an interpretation can be made for control decisions (Morgan et al., 2022).

Fortunately, there is increasing guidance on the use of ruminant parasite diagnostics, from the field to the laboratory (Kaplan et al., 2023; Sabatini et al., 2023).

Diagnostic markers of worm infections have been based on parasitological (e.g., fecal egg count [FEC], coproculture, and Baermannization), immunological (e.g., parasite-specific antibodies in serum or milk), pathophysiological (e.g., ocular mucous membrane color measured by FAMACHA, packed cell volume, plasma/serum pepsinogen, and diarrhea/dag score) and performance-based (e.g., liveweight gain, body condition score [BCS], and milk production) indicators (Charlier et al., 2022). All of these diagnostic tests currently available to veterinarians, advisors, and farmers can potentially be used for deploying the basket of options. However, the decision to use one test or another test will depend on many factors, which may vary by sector, region, and individual farm. FEC, milk ELISA (for detection of *Ostertagia* antibodies in cattle), FAMACHA (for the estimation of the level of anemia associated with *Haemonchus* in small ruminants), diarrhea/dag score, and BCS are the diagnostic markers that best fit into a basket of options for SWC and can be integrated into a sustainable decision support system

On the other hand, diagnostic techniques for AR in ruminants include the FEC reduction test, in vitro tests based on egg hatching or larval development, and DNA-based methods. However, according to the stakeholder consultation during COMBAR, a combination of all these tests was considered needed for addressing AR (reviewed by Charlier et al., 2022).

In recent years, technological innovation has led to an acceleration of diagnostic capabilities. New tests are therefore expected to be developed in the near future but are not yet in the basket. An overarching priority is the availability of pen-side tests and associated decision support tools that provide rapid information on levels of infection and disease. The diagnostic needs include the development of scalable detection methods using artificial intelligence, innovative molecular and proteomic methods for automated parasite egg counting, antibodies and other biomarkers (Rinaldi et al., 2022). Furthermore, next-generation sequencing (NGS) technologies are transforming our understanding of the genetic basis of AR and epidemiological studies of ruminant gastrointestinal parasites. They also have the potential not only to contribute to the development and validation of molecular diagnostic tests but also to be used directly in routine diagnostics by integrating species-specific identification and AR into a single test (Antonopoulos et al., 2024). However, several issues remain to be addressed for the potential of NGS to be fully realized in worm diagnosis. First and foremost, although there are diagnostic markers of benzimidazole and levamisole resistance, there is not yet a marker for macrocyclic lactones, although a strong candidate locus has recently been identified (Doyle et al., 2022). In addition, although the technology is well developed for gastrointestinal nematodes, it has not yet been adapted to the detection of fluke or lungworm, or for AR in these helminths. The second issue is the connection of genotype with phenotype. Most genetic markers identified to date have been initially identified in laboratory-adapted isolates of *H. contortus*, and then validated with a combination of field isolates and functional characterization. This process is slow and labor intensive and until recently there was little alternative. However, in recent years, the adaptation of larval migration assay technology to high-throughput capacity opens the pathway toward large-scale phenotypic characterization of field isolates and the creation of a biobank, which will greatly aid in the development of future molecular diagnostics (Antonopoulos et al., 2024). Projects such as SPARC can directly contribute toward this goal by facilitating researchers interacting directly with farmers and veterinarians through the networks created as part of the project. SPARC will provide an invaluable network for future research on AR, allowing researchers to access field isolates from across Europe, and from a wide range of different farms and conditions.

## Understanding End User Needs

**Figure 4.**
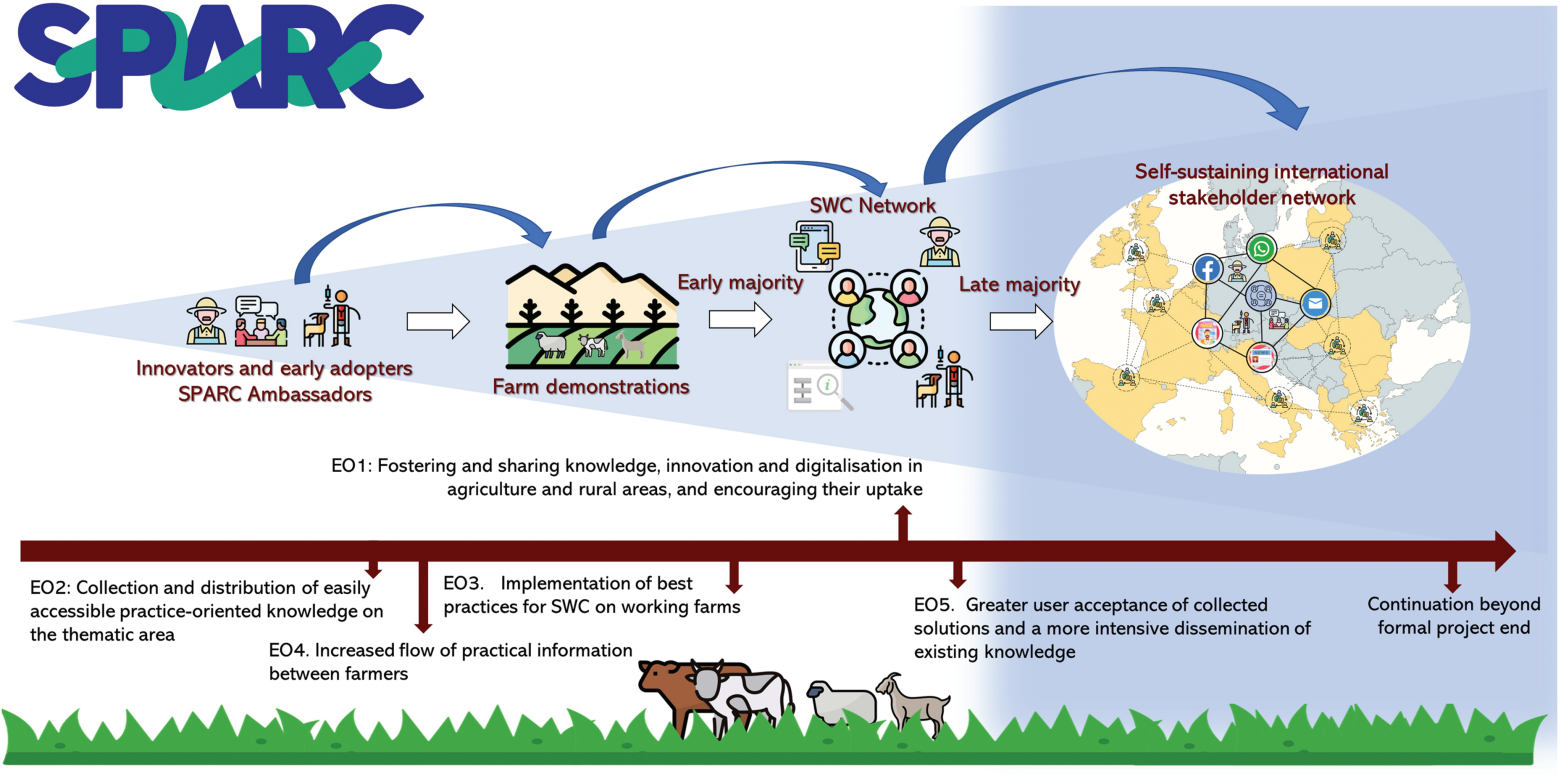
A pathway to implementation of SWC practices across Europe, supported by the thematic network SPARC.

Despite the availability of SWC practices, their adoption by the farmers remains slow and patchy. Several socio-psychological factors affecting farmers’ intention to apply SWC practices have been identified such as the perceived pressure of important referents (e.g., veterinarians and peers), risk perception, and economic and sustainability motives. Additionally, these factors have shown evidence of geographical variation (Vande Velde et al., 2023). This knowledge has now to be deepened in order to develop and disseminate tailored SWC strategies across Europe. Building blocks include (i) prototype decision support tools; (ii) insights into farmer behavior to apply diagnostic tools for worm infections (Vande Velde et al., 2018); (iii) technological opportunities from precision agriculture (data approaches, apps); and (iv) the growing public interest in integrated pest/parasite management combining the reduced use of chemicals with other supportive measures for sustainable parasite control. Thus far, information exchange on SWC has mostly been top-down from knowledge institutes and extension organizations to the farmer. In SPARC, the ambition is to make a change to a participatory approach, where knowledge is built and shared through equal involvement of different stakeholders. As shown in Figure 4, the ambition is to reach innovators and early adopters who can act as ambassadors for SWC. Fueled by farm demonstration and knowledge exchange activities, this can ignite the establishment of local and national networks on SWC. Through the support from an international stakeholder network, we hope that these networks will become self-sustainable beyond the initial EU funding as long as there is a need.

## Conclusion

SWC approaches have been researched to address the escalating problem of AR. While some approaches still need further improvement through research and development such as control through vaccination, several tools and control strategies can be adopted now to reduce the negative impact of worm infection on ruminant health, welfare, and productivity, while at the same time reducing the reliance on anthelmintic drugs. These SWC approaches include the increased use of diagnostics and decision support to enable targeted and selective anthelmintic treatments as well as complementary control measures, including bioactive forages, grazing management, predatory fungi, and selective breeding. The adoption of these practices remains low and this has spurred studies toward the drivers and barriers of SWC behavior. Equipped with the insights from these studies, the time is now ripe for implementing SWC across Europe through participatory approaches and the development of a lasting CoP that involves all stakeholders at local, national, and international levels.

## About the Authors

 **Alistair Antonopoulos** is a scientific project manager at Kreavet, focusing on gastrointestinal nematode parasites of livestock, anthelmintic resistance, epidemiology and biosecurity, and molecular diagnostics. He holds BSc in biomedical sciences from Queen Mary College, University of London, United Kingdom, master’s in infection and immunology, and PhD in veterinary medicine, both from the University of Glasgow, United Kingdom. Alistair previously worked as a postdoctoral research assistant at the University of Glasgow focusing on next-generation sequencing of cattle parasites in Scottish dairy herds, in addition to prior experience in designing molecular diagnostics for dengue virus, and several years of experience in clinical microbiology at the Bristol Royal Infirmary.

 **Dave Bartley** is a principal investigator in the Disease Control Department at the Moredun Research Institute. He obtained his PhD from the University of Edinburgh on the characterization and management of anthelmintic resistance (AR) in United Kingdom sheep nematodes. He has a keen interest in all aspects of livestock nematode research, particularly, in areas around AR, improving diagnostics, sustainable control strategies, and stakeholder engagement. Currently, he has a number of projects including Biosecurity and Roundworm Advice for Cattle Enterprises, transmission risks of animal movement and wildlife in the dissemination of nematodes species, macrocyclic lactone insensitivity in bovine lungworm, and factors that influence farmer’s attitudes and behaviors with respect to nematode control and biosecurity.

 **Johannes Charlier** is a veterinary scientist and founding manager of the Animal Health Research & Consulting Agency, Kreavet. He obtained his PhD in veterinary sciences from Ghent University in 2002 where he specialized in the diagnosis, epidemiology, control, and economics of parasitic infections in ruminants. His research contributions were published in more than 100 scientific publications and were the subject of 2 international awards. Johannes was the chair of the COST Action COMBAR “Combatting Anthelmintic Resistance in Ruminants” (2017 to 2022) and is a project manager of DISCONTOOLS, a coordinator of the secretariat of the STAR-IDAZ international research consortium on animal health, and a technical coördinator of the EU Thematic Network “SPARC—Sustainable Parasite Control in Ruminants”. **Corresponding author:**jcharlier@kreavet.com

 **Edwin Claerebout** graduated as a veterinary surgeon from Ghent University in 1990. After a short stay in a mixed practice in Northern Ireland, he became an assistant at the Laboratory of Parasitology, Faculty of Veterinary Medicine, Ghent University. In 1998, he obtained his PhD in veterinary sciences, on the effect of chemoprophylaxis on acquired immunity to gastrointestinal nematodes in cattle. After 3 yr as a postdoc, he was appointed as a lecturer (assistant professor) at Ghent University in 2001. He became a senior lecturer (associate professor) in 2005 and a full professor in 2010. He is teaching parasitology and parasitic diseases. Since 2003, he is a diplomate of the European Veterinary Parasitology College.

 **Helena Ferreira** obtained her DVM at the Faculty of Veterinary Medicine of the Technical University of Lisbon (Portugal). At Utrecht University (The Netherlands), she obtained her MSc in epidemiology and PhD in epidemiology of African swine fever virus. Afterward, Helena did a postdoc at Plum Island Animal Disease Center (USA), on Foot and Mouth Disease and ASF epidemiology. Subsequently, she worked on swine immunology at GD Animal Health (Netherlands), followed by a technical manager position on R&D on the topic of swine influenza vaccines at IDT (Germany). Afterward, Helena worked at Ghent University (Belgium) focusing on infectious diseases of production animals. Since 2021, Helena has been working at ILVO (Belgium) as a senior researcher in biosecurity, antimicrobial use and resistance in livestock, One health and socioeconomic aspects of veterinary epidemiology. Helena is a diplomate of the European College of Veterinary Public Health.

 **María Martínez-Valladares** has a degree and PhD in veterinary medicine from the University of León, Spain, and is currently a senior scientist of the Spanish National Research Council at the Institute of Mountain Livestock. She is a member of the research group ENTROPIA (tropical and parasitic diseases), where she leads the research line “Control of diseases caused by helminth parasites”. She is a member of the Livestock Helminth Research Alliance (LiHRA). In 2019, she was awarded with the “Peter Nansen Young Scientist Award” by the World Association for the Advancement of Veterinary Parasitology. In 2020, she was admitted to the Spanish Young Academy, being the only veterinarian until now. She is focused on the study of the phenomenon of anthelmintic resistance, including new methods for its early detection and mechanisms involved in its development, and on alternative and sustainable methods for controlling helminth infections.

 **Marcin Mickiewicz** graduated in veterinary medicine from the Faculty of Veterinary Medicine at the Warsaw University of Life Sciences in 2014. He obtained his PhD from the same university in 2021 with the thesis entitled “Epidemiology of drug-resistant gastrointestinal nematode invasions in goats in Poland”. His work provided the first data on the occurrence of anthelmintic resistance (AR) in small ruminant herds in Poland. He currently holds the position of assistant professor at the Division of Veterinary Epidemiology and Economics, Institute of Veterinary Medicine in Warsaw. His scientific work focuses on the diagnosis of AR of parasites in farm animals, with particular emphasis on in vitro tests and the epidemiology of parasitic infections in small ruminants. He is a (co-)author of 85 scientific publications.

 **Eric Morgan** is a professor in veterinary parasitology at Queen’s University Belfast. After graduating in veterinary medicine from the University of Cambridge, he spent periods in veterinary practice and government, before completing his PhD in parasite epidemiology at the University of Warwick and teaching at Bristol Veterinary School for 15 yr. His research interests focus on the effects of climate on parasite transmission and disease. Through participation in many international collaborations and European projects, he seeks to apply knowledge in this area to more sustainable control of helminths in livestock.

 **Natascha Meunier** is a program manager at Animal Health Ireland working on Irish parasite control and beef health programs. She is a veterinarian, completed her MSc (Utrecht University) and PhD (Royal Veterinary College) in epidemiology and is a diplomate of the European College of Veterinary Public Health. Her current research interests include herd health planning, sustainable parasite control, and effective science communication.

 **Laura Rinaldi** (MSc, PhD in veterinary sciences) is a full professor of parasitology and parasitic diseases and deputy dean at the Department of Veterinary Medicine and Animal Production, University of Napoli Federico II, Italy. She has more than 20 yr of experience in the field of parasitic diseases of veterinary and medical importance. Since 2020, she is the designated Director of the WHO Collaborating Centre for Diagnosis of Intestinal Helminths and Protozoa. She is currently a coordinator/principal investigator/work package leader of several national and EU projects. Key research topics include epidemiology (using geospatial tools), diagnosis (improving traditional and using innovative techniques), and control of helminths of veterinary and medical importance. Her main research areas are focused on helminths (e.g., gastrointestinal nematodes and flukes) and anthelmintic resistance in ruminants.

 **Smaragda Sotiraki** is a senior researcher and leads a research group on parasitology and parasitic diseases at the Veterinary Research Institute of the Hellenic Agricultural Research Organization (ELGO-DIMITRA) and focuses her R&D activities on epidemiology of parasitic infections, integrated disease management in livestock (including antiparasitic treatments and alternatives solutions) and spread of parasitic zoonoses. She holds a degree in veterinary medicine and a doctorate in veterinary parasitology, both from the Aristotle University of Thessaloniki, Greece and conducted postdoctoral research for over 2 yr at the Veterinary School in Denmark. She is also a de facto member of the European College for Veterinary Parasitology currently serving as vice president. Dr Sotiraki has over 20 yr R&D and teaching experience in veterinary parasitology and significant managerial experience in research projects having participated in more than 40 major R&D programs at National and International level.

 **Tong Wang** works as an R&D Manager in Kreavet, bringing extensive experience in applied research, predominantly in parasite epidemiology. He earned his PhD in veterinary parasitology from the University of Bristol in 2015. Following his PhD, Tong spent 5 yr as a postdoctoral researcher with Agriculture and Agri-Food Canada and the University of Calgary. His work in the United Kingdom and Canada has involved significant interactions with livestock farmers through planning and conducting field research. Tong has successfully managed multiple research initiatives, consistently delivering high-quality outcomes that have led to numerous peer-reviewed publications and conference presentations. He has a strong background in pasture risk assessment with predictive modeling.
